# Supplementary material for: Development and Validation of the Breastfeeding Literacy Assessment Instrument (BLAI) for Obstetric Women
Source: Int J Environ Res Public Health. 2023 Feb 21;20(5):3808. doi: 10.3390/ijerph20053808 (PMC10000890; doi:10.3390/ijerph20053808)
Supplement: Supplementary file 1 [file ijerph-20-03808-s001.zip › ijerph-2203060-supplementary.pdf]

Supplementary Table S1. Original Spanish and translated versions (not validated) of the Breastfeeding Literacy Assessment Instrument.

**The original version adapted to the sociocultural context of Spain**	English translated version. No linguistic validation was performed.
Acceso	Access
1. Saber a qué profesional (médico/a, pediatra, enfermero/a, matrón/a, asesor/a de lactancia materna) debo dirigirme para resolver un problema que afecta o puede afectar a la lactancia materna (ej. problema de salud materno, medicación materna, problemas con el pecho, etc.)	1. Understand who to contact (such as a doctor, pediatrician, nurse, midwife, or breastfeeding counselor) in order to address and resolve any issues related to breastfeeding (such as a maternal health concern, medication, or breastfeeding difficulties)
2. Ser capaz de expresar de manera clara, a los diferentes profesionales sanitarios, todas aquellas dudas, problemas o preocupaciones relacionadas con la lactancia materna, para que puedan ayudarme.	2. To be able to express clearly to the different health professionals all those doubts, problems or concerns related to breastfeeding so that they can help me.
3. Informarme sobre los recursos relacionados con la lactancia materna que ofrece mi entorno sanitario (ej. Talleres de lactancia materna) y/o mi comunidad (ej. Asociaciones de apoyo a la lactancia materna, Asociaciones de educación maternal)	3. Inform me about the resources related to breastfeeding offered by my healthcare environment (e.g. breastfeeding workshops) and/or my community (e.g. breastfeeding support associations, maternal education associations).
4. Consultar diversas fuentes de información para estar informada y anticiparme a posibles problemas que puedan tener lugar en el transcurso de la lactancia materna.	4. Consult different sources of information to be informed and anticipate potential issues that may arise during breastfeeding.
5. Ser capaz de obtener información de calidad relacionada con la lactancia materna en libros u otro tipo de fuentes de información	5. Being able to obtain quality information related to breastfeeding from books or other sources of information.
6. Buscar información acerca de mis derechos laborales y sociales como madre lactante (ítem eliminado)	6. Seek information about my employment and social rights as a breastfeeding mother (item deleted).
**Comprensión**	**Understand**
1. Entender, cuando leo información sobre lactancia materna, todo el vocabulario de utilizado y comprender a qué hacen referencia cada uno de esos términos (ej. Ingurgitación mamaria, crisis de lactancia materna…)	1. Understand, when I read the information on breastfeeding, all the vocabulary used and understand what each of these terms refers to (e.g. breast engorgement, breastfeeding crisis...).
2. Ser capaz de entender, cuando hablo con un profesional sanitario, todo aquello que me ha explicado en relación con la lactancia materna	2. Be able to understand, when I talk to a health professional, everything that they have explained to me concerning breastfeeding.
3. Preguntar a los profesionales sanitarios en cada visita, todo aquello relacionado con la lactancia materna que no haya comprendido del todo bien	3. To ask the health professionals at each visit about anything related to breastfeeding that I have not fully understood.
4. Comprender los beneficios de la lactancia materna sobre la salud de mi bebé y sobre la mía propia (disminución del riesgo de sufrir ciertas enfermedades, refuerzo del vínculo materno-filial, etc.)	4. To understand the benefits of breastfeeding for my baby's health and my own (reduced risk of certain diseases, strengthening the mother-child bond, etc.).
5. Conocer cómo influyen ciertos hábitos potencialmente nocivos (infusiones, medicamentos, consumo de alcohol, etc.) sobre mi bebé amamantado con lactancia materna	5. To know how certain potentially harmful habits (herbal teas, medicines, alcohol consumption, etc.) affect my breastfed baby.
**Evaluación**	**Appraise**

1. Reflexionar acerca de la veracidad de la información relacionada con la lactancia materna antes de asumirla como verdadera	1. Reflect on the veracity of information related to breastfeeding before assuming it to be true.
2. Saber, en cada momento, el grado de calidad de la información sobre lactancia materna que recibo: a través de recursos online (páginas web, redes sociales, etc.)	2. Know, at any given moment, the quality of the breastfeeding information I receive: through online resources (websites, social networks, etc.).
3. Identificar cuando un “consejo” recibido por parte de personas de mi entorno (familiares, amigos, conocidos) puede repercutir negativamente sobre la lactancia materna	3. Identify when "advice" received from people in my environment (family, friends, acquaintances) could negatively impact breastfeeding.
4. Ser capaz de reflexionar acerca de los consejos relacionados con la lactancia materna, que recibo por parte de personas de mi entorno (familiares, amigos, conocidos) que podrían afectar al éxito de la lactancia materna	4. Be able to reflect on breastfeeding-related advice I receive from people around me (family, friends, acquaintances) that could affect breastfeeding success.
5. Identificar cuando una recomendación sanitaria puede repercutir negativamente sobre la lactancia materna	5. Identify when a health recommendation may have a negative impact on breastfeeding.
6. Ser capaz de preguntar, a un profesional sanitario que me ha aconsejado sobre lactancia materna, todo aquello que me haya generado dudas en base a mi experiencia/conocimientos previos (ítem eliminado)	6. Be able to ask a health professional who has given me advice on breastfeeding about anything that has given me doubts based on my previous experience/knowledge (item deleted).
7. Ser capaz de darme cuenta cuando alguien (profesional sanitario o persona de mi entorno) que me aconseja sobre lactancia materna no está correctamente formado o actualizado en lactancia materna	7. Being able to realise when someone (health professional or person in my environment) who advises me on breastfeeding is not adequately trained or up-to-date in breastfeeding.
8. Ser capaz de darme cuenta cuando mis acciones y/o hábitos influyen, positiva o negativamente sobre el éxito de la lactancia materna	8. To be able to notice when my actions and/or habits influence, positively or negatively, the success of breastfeeding.
9. Valorar cuando sería conveniente solicitar asesoramiento en lactancia materna	9. Assess when it would be appropriate to seek breastfeeding counselling.
10. Saber cuándo mis actos/hábitos/circunstancias influyen en el aumento/disminución de leche materna	10. Know when my actions/habits/circumstances influence the increase/decrease of breastmilk.
**Aplicación**	**Apply**
1. Poner en práctica las recomendaciones sobre lactancia materna, aunque ello implique realizar cambios en mi entorno/hábitos u otras circunstancias, para lograr una lactancia materna exitosa	1. Put the breastfeeding recommendations into practice, even if it means making changes in my environment/habits or other circumstances, to achieve successful breastfeeding.
2. Repetir, sin ayuda, una posición de lactancia materna (balón de rugby, cuna invertida, loba…) que me han aconsejado	2. Repeat, without assistance, a breastfeeding position (rugby ball, inverted cradle, wolf...) that I have been advised to do.
3. Asegurarme si el medicamento que voy a tomarme implica un riesgo para la lactancia materna antes de tomarlo	3. Make sure if the medicine I am going to take poses a risk to breastfeeding before I take it.
4. Detectar y actuar frente pequeñas dificultades que pueden repercutir negativamente en la lactancia materna, antes de que deriven en un problema mayor (agarre incorrecto, ingurgitación mamaria)	4. Identify and address minor difficulties that may negatively affect breastfeeding before they become a larger problem (incorrect latch, engorged breasts).
5. Manejar situaciones relacionados con la lactancia materna (crisis de lactancia materna), aplicando la información de la que dispongo y los recursos disponibles	5. Handle situations related to breastfeeding (breastfeeding crisis), applying the information available to me and the available resources.

6. No poner en práctica “consejos”, relacionados con la lactancia materna, de personas de mi entorno (amigos, familiares, conocidos) sin asegurarme de que la información es correcta.	6. Not to put into practice "advice", related to breastfeeding, from people around me (friends, family, acquaintances) without making sure that the information is correct.
7. Ser capaz de poner en práctica mis conocimientos en lactancia materna para lograr, en la medida de lo posible, aumentar/reducir la producción de leche materna	7. To be able to put my breastfeeding knowledge into practice in order to increase/reduce breastmilk production as much as possible.

Supplementary Table S2. Descriptive and comparative results among Sociodemographic and Variables related to breastfeeding and Access Breastfeeding Literacy.

	Access				p
	Inadequate BFL		Adequate BFL		
	n[1]	%[2]	n	%	
Maternal origin					0.382[3]
Spain	66	38.6	105	61.4	
Rest of the European Union countries	3	33.3	6	66.7	
Center and South America	11	55	9	45	
Other	-	-	1	100	
Age					0.526[4]
18-25 years old	9	47.4	10	52.6	
26-31 years old	26	46.4	30	53.6	
32-37 years old	34	35.4	62	64.6	
38-44 years old	13	39.4	20	60.6	
Educational level					0.844[4]
Primary studies	15	45.5	18	54.5	
Professional training	27	39.7	41	60.3	
Degree	25	36.8	43	63.2	
Master or PhD	15	42.9	20	57.1	
Socioeconomic status					0.016[3]
Low	16	61.5	10	38.5	
Middle	66	37.9	108	62.1	
High	-	-	4	100	
Parity					0.549[4]
First	48	43.6	62	56.4	
Second	26	36.6	45	63.4	
Third or more	8	34.8	15	65.2	
Lactation type					0.054[4]
Exclusive Breastfeeding	55	36.4	96	63.6	
Mixed feeding	9	69.2	4	30.8	
Bottle feeding	18	45	22	55	
Previous Breastfeeding					0.148[4]
Previous Breastfeeding	26	31.7	56	68.3	
No previous breastfeeding	21	47.7	23	52.3	
First pregnancy	33	43.4	43	56.6	
Months EBF previous breastfeeding					0.899[3]
One month or less	6	40	9	60	
2-3 months	-	-	2	100	
4-5 months	3	37.5	5	62.5	
6 months or more	10	33.3	20	66.7	

[1] Absolute frequencies; [2] Relative frequencies; [3] Fisher's exact test; [4] Chi-squared

Supplementary Table S3. Descriptive and comparative results among Sociodemographic and Variables related to breastfeeding and Understand Breastfeeding Literacy.

	**Understand**				**p**
	**Inadequate BFL**		**Adequate BFL**		
	**n[1]**	**%[2]**	n	%	
Maternal origin					0.347[3]
Spain	78	45.6	93	54.4	
Rest of the European Union countries	2	22.2	7	77.8	
Center and South America	10	50	10	50	
Other	1	100	-	-	
Age					0.929[4]
18-25 years old	9	47.4	10	52.6	
26-31 years old	26	46.4	30	53.6	
32-37 years old	41	42.7	55	57.3	
38-44 years old	16	48.5	17	51.5	
Educational level					0.555[4]
Primary studies	15	45.5	18	54.5	
Professional training	32	47.1	36	52.9	
Degree	33	48.5	35	51.5	
Master or PhD	12	34.3	23	65.7	
Socioeconomic status					0.680[3]
Low	13	50	13	50	
Middle	78	44.8	96	55.2	
High	1	25	3	75	
Parity					0.611[4]
First	53	48.2	57	51.8	
Second	30	42.3	41	57.7	
Third or more	9	39.1	14	60.9	
Lactation type					0.023[4]
Exclusive Breastfeeding	61	40.4	90	59.6	
Mixed feeding	10	76.9	3	23.1	
Bottle feeding	21	52.5	19	47.5	
Previous Breastfeeding					0.379[4]
Previous Breastfeeding	32	39	50	61	
No previous breastfeeding	20	45.5	24	54.5	
First pregnancy	38	50	38	50	
Months EBF previous breastfeeding					0.056[3]
One month or less	10	66.7	5	33.3	
2-3 months	-	-	2	100	
4-5 months	4	50	4	50	
6 months or more	9	30	21	70	

[1] Absolute frequencies; [2] Relative frequencies; [3] Fisher's exact test; [4] Chi-squared

Supplementary Table S4. Descriptive and comparative results among Sociodemographic and Variables related to breastfeeding and Appraise Breastfeeding Literacy.

	Appraise				p
	Inadequate BFL		Adequate BFL		
	**n[1]**	**%[2]**	n	%	
Maternal origin					0.229[3]
Spain	62	36.3	109	63.7	
Rest of the European Union countries	1	11.1	8	88.9	
Center and South America	6	30	14	70	
Other	1	100	-	-	
Age					0.871[4]
18-25 years old	6	31.6	13	68.4	
26-31 years old	17	30.4	39	69.6	
32-37 years old	35	36.5	61	63.5	
38-44 years old	12	36.4	21	63.6	
Educational level					0.170[4]
Primary studies	8	24.2	25	75.8	
Professional training	21	30.9	47	69.1	
Degree	24	35.3	44	64.7	
Master or Phd	17	48.6	18	51.4	
Socioeconomic status					1.000[3]
Low	9	34.6	17	65.4	
Middle	60	34.5	114	65.5	
High	1	25	3	75	
Parity					0.011[4]
First	47	42.7	63	57.3	
Second	15	21.1	56	78.9	
Third or more	8	34.8	15	65.2	
Lactation type					0.380[4]
Exclusive Breastfeeding	55	36.4	96	63.6	
Mixed feeding	5	38.5	8	61.5	
Bottle feeding	10	25	30	75	
Previous Breastfeeding					0.022[4]
Previous Breastfeeding	22	26.8	60	73.2	
No previous breastfeeding	12	27.3	32	72.7	
First pregnancy	35	46.1	41	53.9	
Months EBF previous breastfeeding					0.187[3]
One month or less	8	53.3	7	46.7	
2-3 months	-	-	2	100	
4-5 months	3	37.5	5	62.5	
6 months or more	7	23.3	23	76.7	

[1] Absolute frequencies; [2] Relative frequencies; [3] Fisher's exact test; [4] Chi-squared

Supplementary Table S5. Descriptive and comparative results among Sociodemographic and Variables related to breastfeeding and Apply Breastfeeding Literacy.

	Apply				p
	Inadequate BFL		Adequate BFL		
	n[1]	%[2]	n	%	
Maternal origin					0.176[3]
Spain	61	35.7	110	64.3	
Rest of the European Union countries	1	11.1	8	88.9	
Center and South America	5	25	15	75	
Other	1	100	-	-	
Age					0.261[4]
18-25 years old	6	31.6	13	68.4	
26-31 years old	16	28.6	40	71.4	
32-37 years old	31	32.3	65	67.7	
38-44 years old	16	48.5	17	51.5	
Educational level					0.833[4]
Primary studies	9	27.3	24	72.7	
Professional training	23	33.8	45	66.2	
Degree	24	35.3	44	64.7	
Master or Phd	13	37.1	22	62.9	
Socioeconomic status					0.702[3]
Low	8	30.8	18	69.2	
Middle	59	33.9	115	66.1	
High	2	50	2	50	
Parity					0.042[4]
First	43	39.1	67	60.9	
Second	16	22.5	55	77.5	
Third or more	10	43.5	13	56.5	
Lactation type					0.026[4]
Exclusive Breastfeeding	44	29.1	107	70.9	
Mixed feeding	8	61.5	5	38.5	
Bottle feeding	17	42.5	23	57.5	
Previous Breastfeeding					0.159[4]
Previous Breastfeeding	21	25.6	61	74.4	
No previous breastfeeding	16	36.4	28	63.6	
First pregnancy	30	39.5	46	60.5	
Months EBF previous breastfeeding					0.063[3]
One month or less	9	60	6	40	
2-3 months	-	-	2	100	
4-5 months	2	25	6	75	
6 months or more	7	23.3	23	76.7	

[1] Absolute frequencies; [2] Relative frequencies; [3] Fisher's exact test; [4] Chi-squared
